# Comparison of hospitalizations and deaths from COVID-19 2021 *versus* 2020 in Italy: surprises and implications

**DOI:** 10.12688/f1000research.73132.1

**Published:** 2021-09-24

**Authors:** Alberto Donzelli, Marco Alessandria, Luca Orlando

**Affiliations:** 1 Scientific Committee of the Foundation Allineare Sanità e Salute, Milan, Italy, Italy; 2 Dipartimento di Scienze della Vita e Biologia dei Sistemi, Università di Torino, Torino, Italia, Italy; 3 Prima Srl, Agrate Brianza, Italy

**Keywords:** COVID-19 vaccinations; trend of COVID-19 infections, hospitalizations, intensive care admissions, deaths; effectiveness of COVID-19 vaccinations strategies at the community level

## Abstract

Data from the Istituto Superiore di Sanità (ISS) emphasized by the media indicate that COVID-19 vaccination reduces related infections, hospitalizations and deaths.

However, a comparison showed significantly more hospitalizations and intensive care unit accesses in the corresponding months and days in 2021 *versus* 2020 and no significant differences in deaths.

The combination of non-alternative hypotheses may help explain the discrepancy between the results in the entire population and the vaccination’s success claimed by the ISS in reducing infections, serious cases and deaths:

- a bias: counting as unvaccinated also "those vaccinated with 1 dose in the two weeks following the inoculation", and as incompletely vaccinated also "those vaccinated with 2 doses within two weeks of the 2nd inoculation".
- a systematic error: counting as unvaccinated also "vaccinated with 1 dose in the two weeks following the inoculation", and as incompletely vaccinated also "vaccinated with 2 doses within two weeks of the 2nd inoculation".

Many reports show an increase in COVID-19 cases in these time-windows, and related data should be separated

- levels of protective effectiveness in vaccinated people, often considered stable, actually show signs of progressive reduction over time, which could contribute to reducing the overall population result
- unvaccinated people show more severe disease than in 2020, supporting also in humans the theory of imperfect vaccines, which offer less resistance to the entry of germs than the resistance later encountered inside the human body. This favors the selection of more resistant and virulent mutants, that can be spread by vaccinated people. This damages first the unvaccinated people, but ultimately the whole community.

An open scientific debate is needed to discuss these hypotheses, following the available evidence (as well as to discuss the inconsistent theory of unvaccinated young people as reservoirs of viruses/mutants), to assess the long-term and community impact of different vaccination strategies.

## Introduction

The Integrated Surveillance Bulletins of the Istituto Superiore di Sanità (ISS), emphasized by the national media, declare that there has been a sharp decline in infections and contagions, hospitalizations and deaths for this pathology, thanks to the vaccination. The ISS Bulletin of April 30, 2021 states: “The decrease in cases in the older age groups is attributable to the increase in the vaccination coverage in such groups. Starting from the second half of January there is a decreasing trend in the number of cases in healthcare workers and in subjects aged 60 to ≥80 years, probably attributable to the vaccination campaign.”

The campaign began on the 27th December, 2020. As of 28th April, 2021 (the update of the above-mentioned Bulletin), 18,957,365 doses had been administered (13,372,589 first and 5,584,776 second doses). ^1^ On the 7th July, the doses administered had quadrupled, reaching 56,713,862: approximately 91% of >80 year olds in Italy received at least one dose and more than 88% completed the two doses. In the 70–79 years age group, more than 85% has received at least one dose of the vaccine. ^2^ As of the 7th July, 56.7% of the general population had received at least one dose and 37.7% had received full doses. ^3^

## Objective

The aim of this study was to compare the data relating to hospitalizations, access to intensive care unit (ICU) and deaths from COVID-19 in the same period (1st March–7th July) of 2020 and 2021, to highlight the possible impact of COVID-19 vaccine on these outcomes.

## Methods

We downloaded the daily bulletins of the Civil Protection Department (CPD) ^4^; the data are continuously updated (every day positive hospitalized and deceased cases added or removed and corrected on a regional basis).

### Statistic analysis

For the statistical comparison of the data between 1st March–7th July 2020 and 2021 a non-parametric analysis (Mann–Whitney test for independent samples) was used, after performing the Shapiro–Wilk normality test, inasmuch all the data of the variables considered (“Hospitalized with symptoms”, “Intensive care unit” and “Deaths”) do not show a normal distribution. The data were processed using GraphPad Prism 5 software (GraphPad Software, Inc., USA; RRID:SCR_002798), JASP (RRID:SCR_015823) is an open-access alternative software. The significance level “p” was fixed at <0.05.

## Results

**Figure 1.**
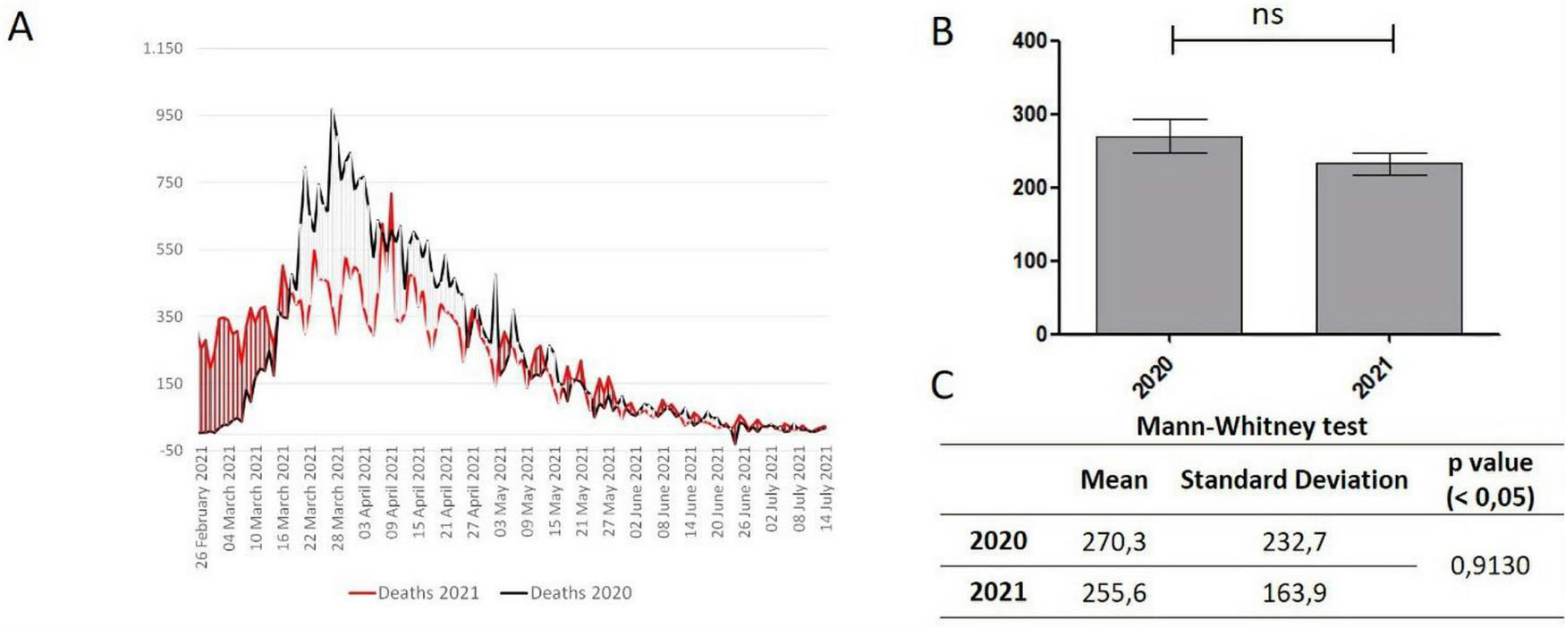
A. Trend of the Deaths variable; B. Significant differences between two periods, with *p < .05; C. Mean, Standard deviation, p value.

No significant variations between 2020 and 2021 were observed in the Deaths variable ( Figure 1).

**Figure 2.**
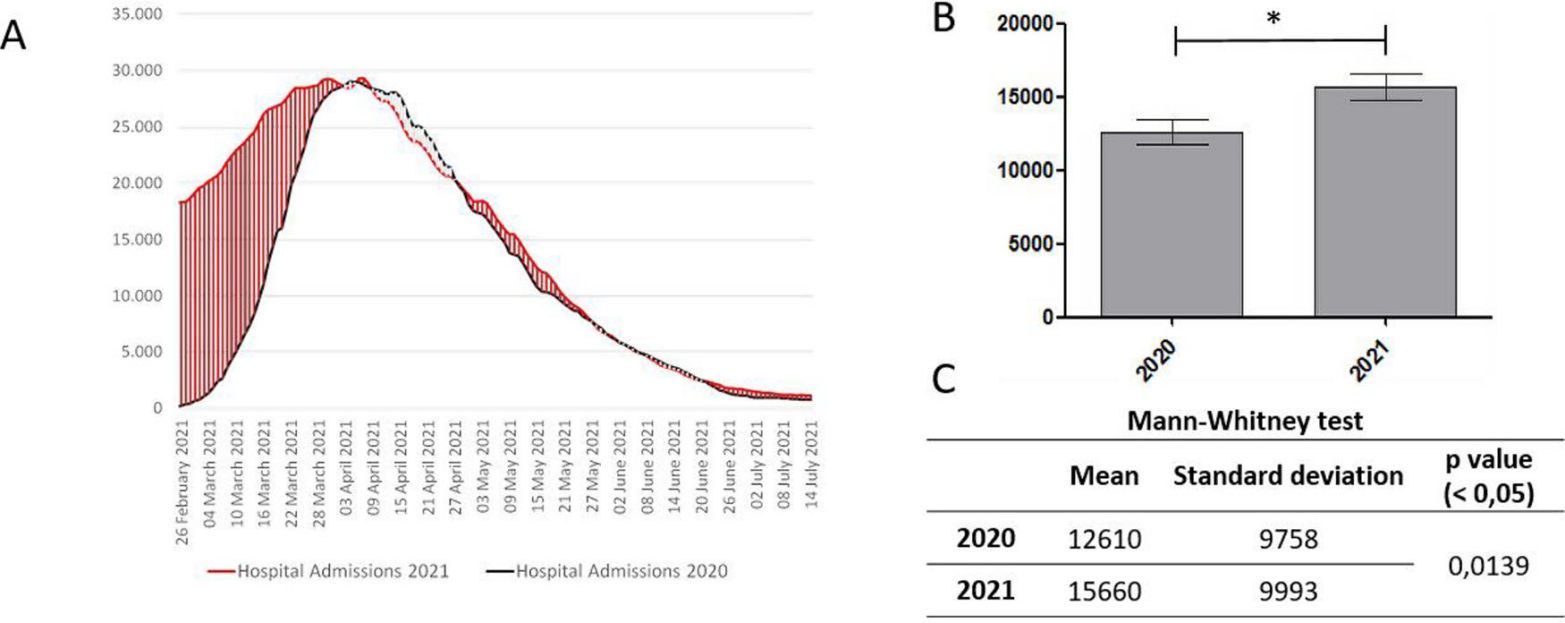
A. Trend of the Hospitalized with symptoms variable; B. Significant differences between two periods, with *p < .05; C. Mean, Standard deviation, p value.

Significant variations between 2020 and 2021 were observed in the Hospitalized with symptoms variable (p = 0.0139; Figure 2).

**Figure 3.**
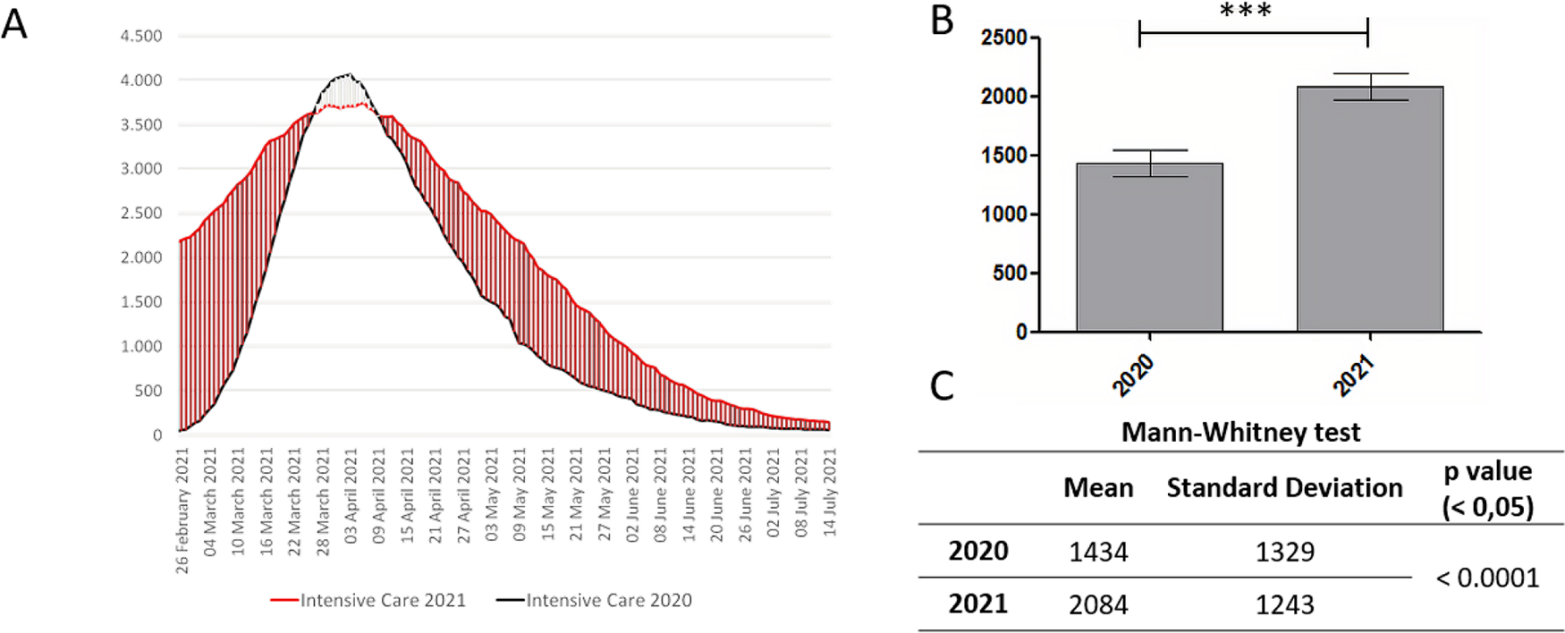
A. Trend of the Intensive care unit variable; B. Significant differences between two periods, with *p < .05; C. Mean, Standard deviation, p value.

Significant variations between 2020 and 2021 were observed in the Intensive care unit variable (p < 0.0001; Figure 3).

## Discussion

The aim of this study was to compare the data of 2020 and 2021 of three variables used by Italian authorities to evaluate the trend of Covid-19 epidemic, to understand the impact of the vaccination.

Data on Deaths ( Figure 1) show that, apart from the mortality peak recorded between the end of March and the beginning of April 2020, the curve from 5th May onwards practically overlaps over the two years, without significant variations.

For Hospitalizations ( Figure 2) the data show, from the end of February to 2nd April, a worse situation in 2021. From the 3rd to the 27th April the two curves had a similar trend, first with fewer beds occupied in 2021, then with a worsening compared with 2020. From 28th April to the end of June, the trend of the curves favored first 2021, then favored 2020 from the 21st June until 7th July, the time limit of the graph. The statistical analysis has shown a statistical variations between two periods.

Also ICU data (fig. 4) showed significant variations between the two periods, with more cases admitted to ICU in 2021 compared with 2020, both from 1st to 21st March and from 10th April to 7th July. The 2020 is worse than 2021 only between 21th March and 10th April: 20 days out of the approximately 120 examined.

The data show a global decrease from the beginning of April, but no improvement in 2021 *versus* 2020. 2021 shows many more accesses in COVID-19 hospital wards than in 2020, without any decrease in specific mortality.

However, for all the three considered variables, the increased access to hospitals in 2021 was more spread over time.

The 2020 restrictions may have had an impact on the distribution of the curve, but it is unlikely that this could have affected the total numbers as well. In fact, restrictions to movement occurred also in 2021: in 2020 they were concentrated in about 2 months (from March to the end of April, up to almost total reopening on 15th June, ^5^ while in 2021 were spread over time, with a mild beginning at the end of October and subsequent tightening and reopening between 26th April and 1st July. ^6^ Moreover, in 2021 there were patchy closures on Italian territory.

The distribution of the two curves is different: the curve of 2020 is distributed over a shorter time period with a slightly higher (and narrower) peak than that of 2021, that appears “spread” over a longer period. Furthermore, the comparison between groups (Mann–Whitney test for independent samples) leaves no doubt about the greater impact of the pandemic in 2021.

Disaggregated data are not available, therefore it is not possible to be more precise from a statistical point of view.

A much better situation could have been expected in 2021, because of the experience in care (including early and home care), and of the vaccination campaign (in the first week of July over a third of the general Italian population received the complete vaccination cycle and over 50% at least one dose.

The decrease in hospitalizations and deaths since the beginning of April was recorded in the same period also in 2020, in a rather faster way, which suggests the contribute of a seasonal effect.

From these observations, the vaccination campaign does not seem to have influenced COVID-19 hospitalizations or deaths overall, in the examined period.

How to reconcile this finding with the ISS Integrated Surveillance Bulletin publications of June 16th and following ^7^ about the success of vaccination in avoiding infections and especially symptomatic, serious cases and deaths? We put forward three hypotheses, which can coexist and explain this apparent paradox.

A) A systematic calculation error may contribute to some extent both to the benefits attributed to the vaccinated and to the harms attributed to the unvaccinated. In fact, the ISS (see *e.g.* the Integrated Surveillance Bulletin from 24th June to 4th July) ^8^ consider “unvaccinated” to be equivalent to “those vaccinated with 1 dose in the two weeks referred to the study” (which according to our calculations amount approximately to 13.5% of the total), and “vaccinated with an incomplete cycle” to be equivalent to “vaccinated with the 2nd dose performed in the two weeks referred to the study” (about 31% of the total). This would not be relevant if the trend of COVID-19 cases in these time windows coincided with the one preceding the inoculations. However, there are many reports that in the days following the inoculations there is an excess of Covid-19 cases. For example, the *BMJ* ^9^ reported a Public Health England study of vaccination in over 70s, which found a “noticeable" increase in COVID-19 infections immediately after receiving the AstraZeneca vaccine. ^10^

Also, a study reported in February on the vaccination program in Israel found a similar spike in cases in newly vaccinated, in this case with Pfizer vaccine, ^11^ with an approximate doubling of the daily incidence after vaccination until about day 8.

The original randomized controlled study (RCT) of Pfizer ^12^ on adults shows in fact a similar effect, with an increase of 40% of cases of “suspected COVID-19” (409 *versus* 287) in the first week in the intervention arm compared with the placebo arm. There is documentation of transient immunosuppression after vaccinations. ^13^ Vaccination with the Pfizer vaccine causes transient drop in lymphocytes over the next 3 days. ^14^ Phase 2 RCTs of AstraZeneca also showed a drop in neutrophils; ^15^ other vaccinations have also shown a fall of neutrophils ^16^ and lymphocytes. ^17^

Therefore, vaccinated people can temporarily develop more cases and infect more, for at least a week after the inoculation and, in that week, the virus multiplies in vaccinated people who develop COVID-19.

If there is an increase in COVID-19 cases in the time window of 14 days following an inoculation and they are counted among the “unvaccinated”, the latter are burdened with an undue excess of cases, simultaneously relieved by “vaccinated”. Therefore, the ISS calculations should be redone, at least keeping the subjects who have received a dose of vaccine in the previous two weeks as separate from the “unvaccinated" and “incompletely vaccinated”.

B) The vaccine effectiveness after the aforementioned 14 days were believed to be quite stable and durable, while now there is evidence of a progressive deterioration in the protective effectivenss of vaccines. This seems plausible, in view of the marked decline in antibody levels measured at various time intervals (14–20 days, 21–41, 42–55, 56–69 and 70 or more) after the second vaccination, ^18^ and as reported by the Israeli Government, ^19^ finding a significant decline in the vaccination effectiveness over the following months.

This could help to explain the results of the overall comparison between hospitalizations and ICU accesses in 2020 *versus* 2021. In fact, the ISS comparisons show important differences in the outcomes between unvaccinated and vaccinated, but do not exclude a progressive reduction of protective effectiveness also in the vaccinated, influencing the overall result on the whole population. ^10^

The ISS data show that symptomatic serious cases would largely be ascribed to unvaccinated (or vaccinated contracting the disease within 14 days of inoculation of the first dose), and it is unlikely that these results can change substantially even applying the corrections suggested in point A). This observation outlines a scenario in which the infection would have moved from a pool of subjects potentially capable of developing severe COVID-19 corresponding to the majority of the Italian population (excluding those who have overcome the infection) to the smaller pool of the “unvaccinated”. If this was true, it would mean that the latter, having reached absolute numbers of deaths comparable to those of 2020, and even greater numbers of hospitalized and ICU patients, would get Covid-19 with more severity than 2020. If so, the hypothesis that the vaccine can protect both those who receive it and *others* should be questioned: instead, it could pose additional risks in the unvaccinated. This may be more than just an hypothesis, already advanced in 2001, ^20^ confirmed experimentally in 2015 for other vaccines ^21^ and proposed again today with possible reference to SARS-CoV-2 vaccines. ^25^ It is a problem occurring if vaccines are *leaky*, ^21^ ^,^ ^22^ when they block the virus entrance door less than they fight the virus inside the body, as in fact is the case with today SARS-CoV-2 vaccines; it is likely that the natural infection (starting from the respiratory tract and not with an injection) helps even better to reduce subsequently the entry of viruses. As a result, the virus multiplies to some extent inside the organism, vaccine antibodies neutralize the vast majority of the viruses but, as unintended effect, they favor precisely those random mutations (and variants) that give the virus greater ability to escape from antibodies or to resist their attack, becoming more virulent. Consequently, some of the vaccinated people could spread more virulent viruses, harming the remaining community (and in the long term probably even the vaccinated, as immune protection tends to decline). Experiments with chickens vaccinated and infected with Marek virus (not deliberately replicable in humans, but which could be replicated in farmed mammals susceptible to SARS-CoV-2, such as ferrets) ^21^ have shown clearly this effect, which harmed mostly the unvaccinated members of the farm, who developed a more serious disease.

### In the face of this possibility, what strategies should be adopted?

First of all, it should be accepted to discuss it seriously, comparing the scientific evidence available for or against this explanatory hypothesis. If this hypothesis is confirmed, a discussion should be opened urgently about how to improve strategies at the population level.

Of course, a possible strategy is that pursued today in Italy and in most of the world: to accelerate the push for universal vaccination, using a mix of obligations, incentives and penalties, up to the complete discrimination of those who do not adhere, hoping to end the pandemic, as well as to protect those at risk. This strategy is what the national authorities have chosen so far and of which the majority of the population is convinced. However, part of the population do not accept this strategy (also because dubious constitutional legitimacy). Against such strategy there are:

• the costs, including organizational ones, of universal immunization (worldwide it would involve 8 billion people, in addition to the management of the animal reservoirs already documented ^23^); and many authorities now hypothesize to use periodic boosters;
• the total and severe adverse reactions already known, of the extent of which there is, however, little awareness ^24^ ^,^ ^25^; and the serious adverse effects known or still unknown;
• the highly questionable relationship between expected benefits and risks of vaccinating young people and children, who are offered moreover a vaccine calibrated on the original viral strain, while the new variants are sometimes already called “another virus”;
• the enormous loss of opportunity-cost for the equivalent amount of resources diverted from other health uses.

An alternative could be to focus vaccination on those who can benefit most (elderly, multi-pathological subjects or workers at high professional risk), and, after having protected them, not to promote vaccination towards those who have little to earn from it. Note, for example, that in Italy in 2020 the 0–49 years old population showed a paradoxical reduction of 8.5% in mortality, compared to the average of the five-year period 2015–2019. ^26^ In the first months of 2021 the mortality reduction under the age of 50 years was even more pronounced compared to the five-year reference period (January 2021: −12%; February: −17%, March: −10.8%). ^27^

• Even more so a focused vaccination should apply to young people 0−19, who show a prevalence of asymptomatic cases >50%, paucisymptomatic >30%, and mild cases <20% in the update of mid-July. ^28^ They can help mitigate the epidemic, ^a^ progressively transforming it into a milder endemic, ^35^ ^,^ ^36^ by developing a natural immunity, which seems robust and lasting, as far as we know. ^29^ ^–^ ^32^

Children and adolescents in particular have been shown ^37^ to be less infected and infectious, to have asymptomatic infections much more often than adults and, even when asymptomatic, to develop robust humoral immune responses, persisting for at least one year. ^37^ The argument that, before being naturally immunized, they would constitute a “dangerous reservoir” of viruses seems to have little scientific and logical support. ^a^

## Conclusions

Repeating that this year “in Italy the overall drop in hospitalizations, IT and deaths is due to the vaccine” is not supported by the available data, since the respective trend was even better in 2020, without the vaccine.

The interpretation of this finding should be subject to an open and uncensored scientific debate, aimed at understanding the impact of vaccination strategies also at the global level of the population and in long-term scenarios, as a prerequisite for rational health policy choices, that optimize health outcomes (and sustainability) for the individuals and the community.

## Data availability

### Underlying data sources

https://github.com/italia/covid19-opendata-vaccini - Open Data on delivery and administration of COVID-19 vaccines in Italy.

https://www.epicentro.iss.it/coronavirus/bollettino/Bollettino-sorveglianza-integrata-COVID-19_7-luglio-2021.pdf - COVID-19 epidemic National update July 7, 2021-12 noon (I.S.S.).

https://github.com/pcm-dpc/COVID-19/blob/master/dati-andamento-nazionale/dpc-covid19-ita-andamento-nazionale.csv - Protezione Civile data table.csv used for the development of the comparison graphs 2020 versus 2021.

https://covid19.zappi.me/table/ - Protezione Civile data table (simple format, same data as the previus.csv file) used for the development of the comparison graphs 2020 versus 2021.

https://www.ilpandacentrostudio.it/vaccini.html - Calculations by Antonio Caramia on data from the Extraordinary Commissioner Covid-19 (Dashboard Vaccines).

https://opendatadpc.maps.arcgis.com/apps/dashboards/b0c68bce2cce478eaac82fe38d4138b1 - Dashboard COVID-19 Protezione Civile.

https://www.epicentro.iss.it/coronavirus/bollettino/Bollettino-sorveglianza-integrata-COVID-19_16-giugno-2021.pdf - COVID-19 epidemic National update June 16, 2021-12 noon (I.S.S.).

https://www.epicentro.iss.it/coronavirus/bollettino/Bollettino-sorveglianza-integrata-COVID-19_23-giugno-2021.pdf - Epidemia COVID-19 COVID-19 epidemic National update June 23, 2021-12 noon (I.S.S.).

https://datadashboard.health.gov.il/COVID-19/general - Corona virus in Israel – general situation Dashboard.

## Footnotes

^a^
The issue continuously put forward that unvaccinated children and young people are “a reservoir” of “viruses that continue to circulate” and that “consequently mutate”, actually has limited scientific evidence (and the situation of the vaccinated may not be so different from unvaccinated). This can be explained in three steps:

1) The contagiousness of adults who become infected lasts on average 7 days from the onset of symptoms plus ~2 days before symptoms. A systematic review of 79 studies ^33^ never found live, viable viruses after the 9th day in any of the studies, even if the emission of RNA fragments could sometimes lasts weeks.
  In children, often asymptomatic or paucisymptomatic, the duration of contagiousness seems lower than that of adults: ^33^ therefore it can be estimated in about a week (or a little more)
2) There are 52 weeks in a year. If a child is not infected, she/he is never contagious, nor can be a “reservoir”. If infected, she/he can infect (usually less than adults ^34^) for about a week, and for the other 51 weeks of the year the child has become immune, so not constituting any “reservoir”.
3) The hypothesis A, explained in the discussion above, reports many examples where the vaccinated, in the days following the inoculation, developed more cases of COVID-19 than in the previous period, so resulting more contagious, with a temporary viral multiplication (due to infection pre-inoculum kept under control by innate defenses? Or to infection acquired because of relaxed precautions? Or by temporary immunosuppression?), with some analogies with what follows to natural infection. If the latter is symptomatic, however, the patient becomes aware of it and can isolate himself, while the vaccinated may lack the awareness of a transient potential greater infectivity.
