# Supplementary material for: Comparison of hospitalizations and deaths from COVID-19 2021 versus 2020 in Italy: surprises and implications
Source: F1000Res. 2021 Sep 24;10:964. [Version 1] doi: 10.12688/f1000research.73132.1 (PMC8596176; doi:10.12688/f1000research.73132.1)
Supplement: Peer review history

# Peer review: Reviewer response for version 1

**DOI:** 10.5256/f1000research.76758.r97925

**Published:** 2021-11-16

**Authors:** Marco Cosentino

**Affiliations:** 1 Center for Research in Medical Pharmacology, University of Insubria, Varese, Italy

By use of publicly available data, this study examines SARS-CoV-2 hospitalizations, ICU use and deaths in Italy in 2020 (before mass vaccinations campaign took place) and in 2021, after most of the Italian population received the COVID-19 vaccines. Conclusions challenge the official version that COVID-19 vaccination reduces related infections, hospitalizations and deaths. Indeed, with a very simple and effective approach the study shows that between 2020 and 2021 deaths are substantially the same, while hospitalizations and ICU use are substantially increased.

 The authors provide as possible explanation for the bias in counting as unvaccinated also "those vaccinated with 1 dose in the two weeks following the inoculation", and as incompletely vaccinated also "those vaccinated with 2 doses within two weeks of the 2nd inoculation", and the systematic error due to counting as unvaccinated also "vaccinated with 1 dose in the two weeks following the inoculation", and as incompletely vaccinated also "vaccinated with 2 doses within two weeks of the 2nd inoculation".

 It is however unclear how, and to what extent, misclassification of vaccinated/unvaccinated people would affect results which regard the whole population.

 Indeed, in 2020 it can be assumed that no one was vaccinated, which in 2021, an increasing fraction of people got the vaccine. Would it be possible to separate hospitalizations, ICU use and deaths thus comparing unvaccinated and vaccinated? Anyway, it is my opinion that even without this distinction deaths are the same and are therefore expected to have been not so much affected by mass vaccination campaign. On the other side, increased hospitalizations in early 2021 and increased ICU use in both early and late 2021 m9ght possibly suggest any detrimental effect of vaccine of health status leading to hospitalization.

 Finally, what about criteria for linking hospitalizations, ICU use and deaths to COVID-19? Were they the same throughout all the study? Or did they change and in case to what extent?

Is the work clearly and accurately presented and does it cite the current literature?

Yes

If applicable, is the statistical analysis and its interpretation appropriate?

Yes

Are all the source data underlying the results available to ensure full reproducibility?

Yes

Is the study design appropriate and is the work technically sound?

Yes

Are the conclusions drawn adequately supported by the results?

Yes

Are sufficient details of methods and analysis provided to allow replication by others?

Yes

Reviewer Expertise:

Medicine, Pharmacology, Immunology, Neuroscience

I confirm that I have read this submission and believe that I have an appropriate level of expertise to confirm that it is of an acceptable scientific standard.

# Peer review: Reviewer response for version 1

**DOI:** 10.5256/f1000research.76758.r98994

**Published:** 2021-11-08

**Authors:** Herald Walach

**Affiliations:** 1 Department of Pediatric Gastroenterology and Metabolic Diseases, Poznań University of Medical Sciences, Poznan, Poland

**Review of F1000 MS by Donzelli, Alessandria & Orlando “ *Comparison of hospitalizations and deaths from COVID-19 2021 versus 2020 in Italy: surprises and implications*”**

 Harald Walach

 This is an interesting secondary analysis of publicly available data in Italy on deaths by Covid19, hospitalizations and ICU usage due to this disease, comparing years 2020 and 2021 during the same time window (end of February to beginning of July). The data were compared with robust and very simple statistical methods (non-parametric tests) and yielded significant differences for hospitalizations and ICU usage. The fact that a comparatively robust and insensitive statistical model found such strong effects shows that these effects are indeed substantial.

 This result contradicts official statements that the vaccination campaign in 2021 has reduced the case load, mortality and hospitalizations. The authors produce a succinct argument explaining this discrepancy: it is likely due to the definition used by authorities (actually copied from pivotal regulatory trials) that counts people as vaccinated only after a certain time window after first and second vaccinations. The authors of this paper make a point that it is precisely during this time that vaccinated people are vulnerable or more likely to develop the disease, which would escape the official statistics due to the definition employed.

 The data speak a clear language, and the authors do a good job interpreting and explaining the finding and I think this paper is essentially a valid representation of the situation.

 The authors miss one potentially important point in their explanation: if the Covid-19 vaccines are not as safe as claimed and reports about the side effects as published by Rose (1), which are likely on the low side, are true, then some of the deaths and hospitalizations might be also induced by the vaccination campaign. I think this is a potentially additional factor, but I leave it at the discretion of the authors, whether they want to include this.

 Otherwise, I think this paper is fit for publication. Below are a few points for clarification that I would recommend to make in a revised version:

*Major Points:*

 Since “Death from Covid19” (and hospitalization and ICU usage) is one of the major endpoints of this study, it would be good for readers to know:

1. How this is defined in Italy and
2. Whether this definition has changed between 2020 and 2021,

Just to make sure that any changes potentially visible are not due to changes in definition.

**Minor Points:**

 Abstract: The term “non-alternative hypotheses” is not clear. Perhaps explain or change.

 Abstract: the points “bias” and “systematic error” repeat the same information; perhaps it would be better to say “bias or systematic error” and then bring the information just once.

 Discussion 3 ^rd^ para: “The statistical analysis has shown a statistical *variations* between two periods.” should read “variation” (sing.).

 Discussion, p. 5, middle: “which suggests the contribute of a seasonal effect.” Should read … *contribution*….

 Discussion, p 5, penultimate para: reformulate; this is a bit awkward (for instance: “Vaccinated people are more liable to develop the disease during a short period of time and are more likely to infect others for at least a week…”

 Discussion, p5, last para: “the latter are burdened with an undue excess of cases, simultaneously relieved by “vaccinated”“ This is confusing. Please reformulate, for instance: “… more cases are unduly registered in the group of the “vaccinated”. At the same time, the same number of cases is arithmetically deducted from the group of the vaccinated. This yields the impression that more cases are seen in the group of the unvaccinated than in the group of the vaccinated, which is in fact an error due to case definition.” Or something similar that makes the underlying meaning clearer.

 Consider putting the information hidden in note a) into the text, if no editorial policy or limit in word length speaks against it.

**References and Data-Availability:**

 Perhaps put the links of the data sources behind the references so that it is clear which data-source refers to what point mentioned in the text.

 “Statistic Analysis” should be “Statistical Analysis”

 Figure 1: in the legend, and perhaps in the text, make clear that this is “Death due to Covid19”; the same for the other variables and Figures: make clear that this is Hospitalizations due to Covid19”

 Results: I am not sure that “variation” is the right term here. What was observed was not only a change in variation, but a change of overall magnitude in the number of hospitalization and ICU treatments

 Reference

 Rose, J. (2021). A report on the U.S. vaccine adverse events reporting system (VAERS) on the Covid-19 messenger ribonucleic acid (mRNA) biologicals. *Science, Public Health Policy, and the Law, 2*, 59-80.

Is the work clearly and accurately presented and does it cite the current literature?

Yes

If applicable, is the statistical analysis and its interpretation appropriate?

Yes

Are all the source data underlying the results available to ensure full reproducibility?

Yes

Is the study design appropriate and is the work technically sound?

Yes

Are the conclusions drawn adequately supported by the results?

Yes

Are sufficient details of methods and analysis provided to allow replication by others?

Yes

Reviewer Expertise:

psychology, study design, epidemiology, research methods

I confirm that I have read this submission and believe that I have an appropriate level of expertise to confirm that it is of an acceptable scientific standard.
